# Evaluating Cadaveric Simulation Versus Traditional Instruction for Teaching Ultrasound-Guided Serratus Anterior Plane Blocks: Effects on Procedural Competency

**DOI:** 10.7759/cureus.99059

**Published:** 2025-12-12

**Authors:** Alexandra E Taylor, Anatalia Kerstan, Ashley Hoover, Mahnoor Malik, Austin Mondello, Sarah Boulos

**Affiliations:** 1 Medicine, Rocky Vista University College of Osteopathic Medicine, Englewood, USA; 2 Emergency Medicine, Rocky Vista University College of Osteopathic Medicine, Parker, USA

**Keywords:** anesthesia education, cadaveric model training, hands-on training, medical education, medical students, procedural competency, regional anesthesia, serratus anterior rib block, training effectiveness, ultrasound-guided blocks

## Abstract

Background: Medical education often relies on lecture-based instruction and videos to teach procedural skills. However, cadaveric-based training offers a more hands-on approach that may enhance procedural competency. This study evaluates the efficacy of cadaveric model training in teaching medical students the serratus anterior plane block (SAPB) compared with traditional lecture- and video-based instruction.

Objective: The objective of this study is to compare the procedural competency of medical students trained using a traditional method (lecture + video) versus those receiving additional cadaveric model practice. We hypothesized that cadaveric training would lead to greater proficiency in performing the SAPB.

Methods: A randomized comparative study was conducted with 26 medical students assigned by computer-generated allocation to either a traditional instruction group (lecture + video; n = 13) or an intervention group (lecture + video + cadaveric training; n = 13). All participants completed a pre-study knowledge survey followed by standardized instruction. The intervention group additionally received hands-on cadaveric practice. Within one week, all students completed a 32-point procedural competency assessment and a post-study knowledge survey. Knowledge scores and procedural performance were compared between groups using independent samples t-tests.

Results: The intervention group demonstrated significantly higher procedural competency scores than the traditional instruction group, particularly in key steps such as transducer positioning, needle insertion, and injection site preparation. Both groups showed similar improvements in written knowledge (pre- vs. post-test), with no significant difference between groups (p > 0.05). In contrast, procedural performance scores were significantly higher in the cadaveric training group (p < 0.05).

Conclusion: Cadaveric model training significantly enhances procedural competency in medical students learning the SAPB compared with traditional lecture- and video-based instruction. While written knowledge gains were comparable, hands-on cadaveric experience provided a key advantage in developing clinical procedural skills. These findings support incorporating cadaveric-based instruction into procedural skill curricula for improved bedside preparedness.

## Introduction

Traditional medical education often relies on lectures, readings, and demonstration videos to teach procedural skills [1]. Although these methods efficiently convey cognitive information, they do not replicate real clinical environments or provide the tactile experience necessary for developing procedural proficiency. Ultrasound-guided regional anesthesia (UGRA) requires not only anatomical knowledge but also coordinated psychomotor skills and real-time image interpretation [2]. Cadaveric models offer an opportunity to practice these integrated skills by providing anatomic realism and tactile feedback that cannot be achieved through passive learning modalities [3].

The serratus anterior plane block (SAPB) is an ultrasound-guided technique commonly used to provide analgesia for rib fractures, thoracic surgery, and chest wall trauma [4,5]. Effective performance of SAPB can reduce pain, improve ventilation, and decrease opioid requirements, making early student exposure valuable for clinical readiness across multiple specialties.

Although cadaveric simulation is widely recognized as beneficial for teaching anatomy and improving learner confidence, few studies have evaluated its direct effect on measurable procedural competency, particularly for SAPB. Existing UGRA education literature often emphasizes learner satisfaction or confidence rather than objective performance outcomes. This study addresses this gap by comparing traditional instruction (lecture + video) with an added cadaveric training component to determine whether cadaveric practice improves procedural competency in performing SAPB.

## Materials and methods

Study design and setting

This randomized, comparative educational study was conducted at Rocky Vista University College of Osteopathic Medicine (RVUCOM) in Colorado. All instructional activities and assessments took place in the on-campus anatomy laboratory and classroom facilities over a 1-2 week period. A total of 26 medical students participated in the study.

A formal power calculation was not performed; the sample size reflects a convenience sample typical of pilot educational research.

Participants and inclusion/exclusion criteria

Eligible participants were RVUCOM medical students aged 18 years or older who voluntarily consented to participate and were comfortable working in a cadaveric environment.

Inclusion Criteria

RVUCOM medical students aged ≥18 and those willing and able to provide informed consent were included in the study.

Exclusion Criteria

Non-medical students, individuals uncomfortable with cadaveric procedures, participants under age 18, incomplete completion of pre-study assessments, and latex-allergic students (all materials were latex-free) were excluded.

A total of 26 students were assessed for eligibility; all 26 met inclusion criteria, consented to participate, were randomized (13 per group), and were included in the final analysis with no exclusions or loss to follow-up.

Group allocation and confidentiality

Following consent, students provided contact information for scheduling. Each participant received a confidential study ID and was randomly assigned using computer-generated allocation to either the Traditional Instruction Group (lecture + video; n = 13) or the Cadaveric Training Group (lecture + video + cadaveric practice; n = 13).

No blinding of participants or assessors occurred due to the nature of the educational intervention, which is consistent with simulation-based educational RCTs.

Only investigators had access to the secure linkage between participant identities and study IDs. All data were stored in encrypted, password-protected files accessible only to the research team.

Instructional intervention

All participants completed a Pre-Study Knowledge Survey (12-point scale) evaluating baseline understanding of SAPB anatomy and technique. The pre- and post-study surveys assessed factual procedural knowledge only; confidence or self-efficacy was not measured. No validated SAPB-specific knowledge assessment instrument exists; therefore, the survey items were developed by faculty content experts based on established procedural steps and educational objectives.

Traditional Instruction Group (Control)

A 30-45 minute faculty-led lecture covering SAPB anatomy, indications, and procedural steps and a 15-minute instructional video demonstrating ultrasound-guided SAPB was given.

Cadaveric Training Group (Intervention)

The Cadaveric Training Group received all control group instructions, additionally participated in a supervised live demonstration on a cadaver, and performed hands-on ultrasound scanning and needle-tracking practice.

All participants completed a Post-Study Knowledge Survey (12-point scale) and a procedural competency assessment, performed 5-7 days after training to ensure a standardized consolidation interval.

Procedural competency assessment

SAPB procedural competency was evaluated using a 32-point checklist adapted from StatPearls procedural guidelines [2]. Assessed elements included: Proper positioning; ultrasound probe placement at the fifth rib in the mid-axillary line; needle insertion with continuous visualization; identification of correct fascial layers; injection technique and documentation

Each completed task earned one point (maximum score: 32). A score ≥26/32 (80%) was considered proficient.

Although the checklist has not undergone formal reliability testing (e.g., Cronbach’s alpha), it is derived from published procedural steps and demonstrates strong face validity for assessing novice learners in UGRA education.

Data collection and storage

All surveys and procedural scores were recorded using de-identified study IDs. A fully anonymized dataset was used for analysis. Identification keys were stored separately on password-protected systems accessible only to the investigative team.

Statistical analysis

Data were assessed for normality using the Shapiro-Wilk test. Independent (Welch’s) two-tailed t-tests were used to compare post-instruction knowledge scores and procedural competency scores between groups, with α = 0.05. Pre- to post-knowledge score changes were also calculated within each group. Effect sizes (Cohen’s d and Hedges’ g) were computed. Descriptive statistics included means, standard deviations (SD), and 95% confidence intervals. All analyses were conducted in Python.

Welch’s t-test was selected due to unequal variances between groups, providing a more robust comparison under conditions of heteroscedasticity.

Ethical considerations and safety protocols

The study was approved as minimal-risk educational research under institutional guidelines. All participants provided written informed consent. No live patients or PHI were involved. Anatomy laboratory safety standards were followed, including PPE use and formaldehyde exposure mitigation. Pregnant or breastfeeding individuals were provided respirators upon request.

## Results

Participant characteristics

A total of 26 medical students completed all study components, with 13 assigned to each instruction group. All participants were first- or second-year medical students, and the distribution of class year was similar between groups. Prior ultrasound familiarity (self-reported exposure level) was also comparable across groups. No statistically significant baseline differences were identified.

Written knowledge

**Figure 1:**
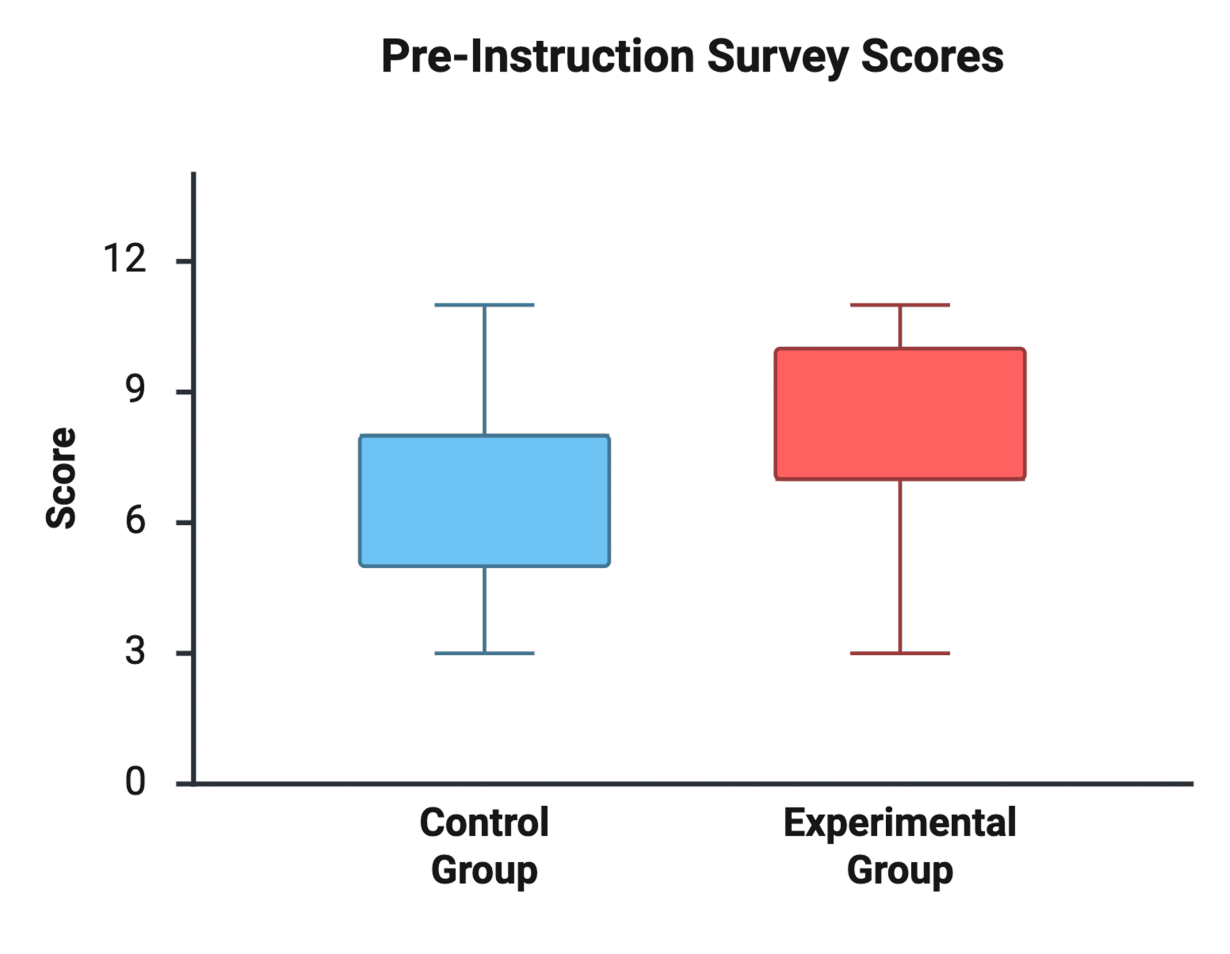
Pre-instruction Survey Scores Comparison of pre-instruction written knowledge scores between the instructional groups (out of 12 possible points).

**Figure 2:**
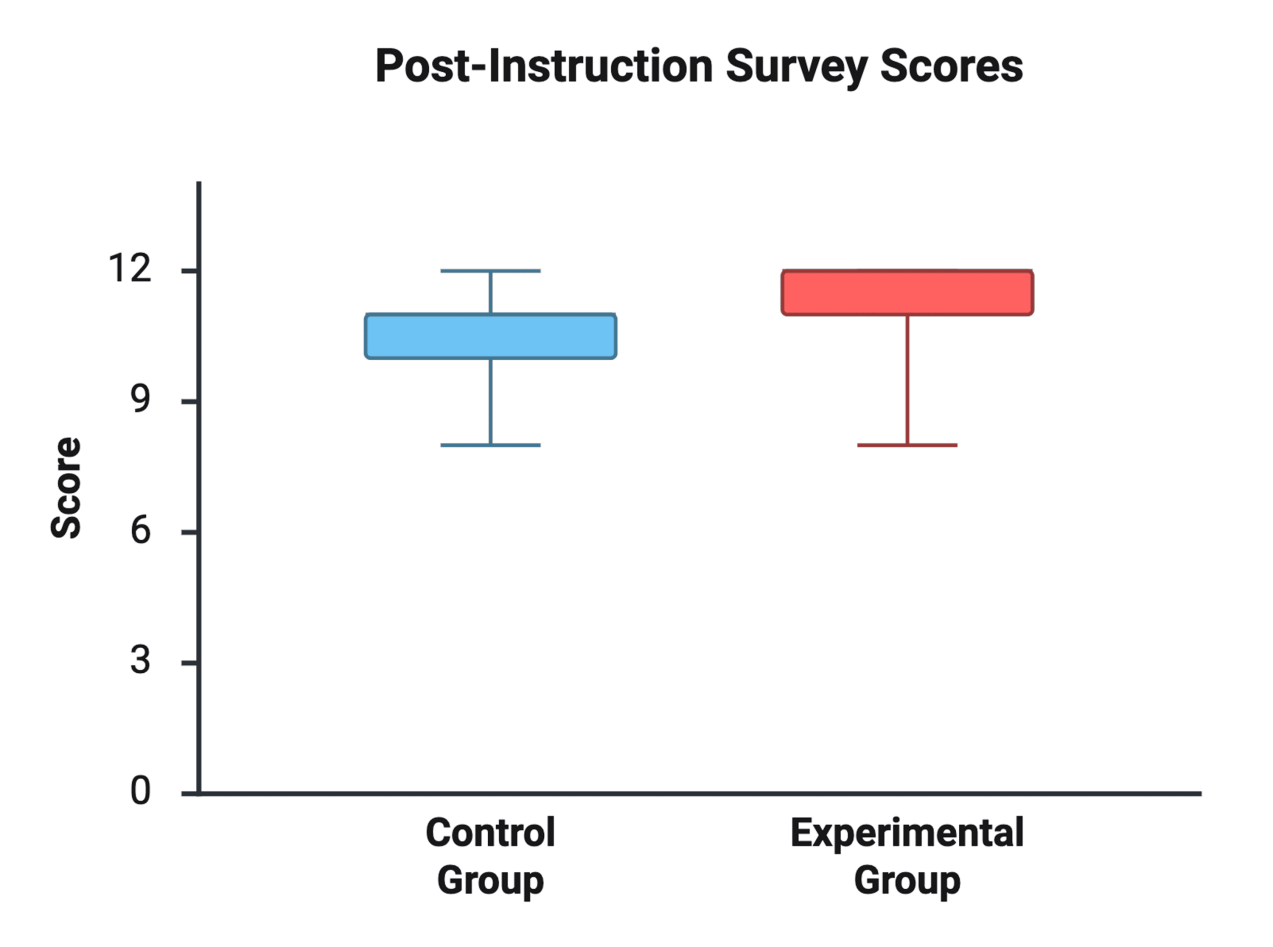
Post-instruction Survey Scores Comparison of post-instruction written knowledge scores between the instructional groups (out of 12 possible points)

Pre-instruction survey scores were not significantly different between the Traditional Instruction Group and the Cadaveric Training Group (6.92 ± 2.36 vs. 7.85 ± 2.30 out of 12, respectively; p = 0.32). After instruction, both groups demonstrated improvement in written knowledge. Post-instruction scores were 10.23 ± 1.24 in the traditional group and 11.15 ± 1.28 in the cadaveric group, with a mean difference of 0.92 points (95% CI: −0.10 to 1.94; p = 0.074) (see Figures 1, 2).

Procedural competency

**Figure 3:**
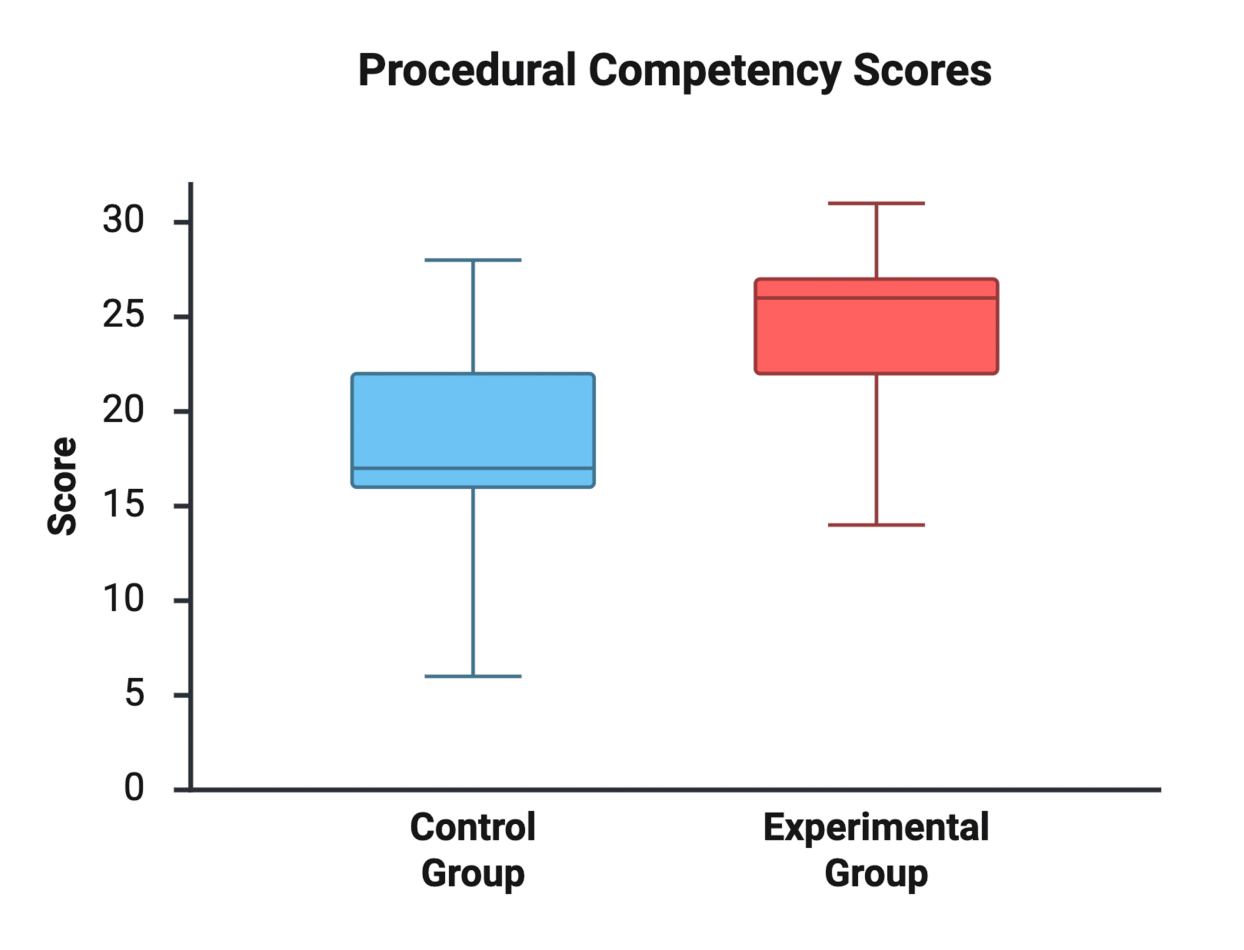
Procedural Competency Scores Comparison of serratus anterior plane block procedural scores between instructional groups (out of 32 possible points).

The Cadaveric Training Group scored significantly higher on the procedural competency checklist compared to the traditional group (24.38 ± 4.61 vs. 18.31 ± 5.59 out of 32, respectively). The mean difference was 6.08 points (95% CI: 1.92-10.23; p = 0.006), with a large effect size (Hedges’ g = 1.15) (see Figure 3 and Table 1).

**Table 1:** Summary of Knowledge and Procedural Competency Scores Values shown as mean ± standard deviation. p < 0.05 considered statistically significant.

Outcome	Group	Mean ± SD	95 % CI	Welch t	p value	Hedges g
Pre-test (0–12)	Control	6.92 ± 2.36	5.50 – 8.35	1.01	0.32	0.38
	Experimental	7.85 ± 2.30	6.45 – 9.24			
Post-test (0–12)	Control	10.23 ± 1.24	9.48 – 10.98	1.87	0.074	0.71
	Experimental	11.15 ± 1.28	10.38 – 11.93			
Procedural competency (0–32)	Control	18.31 ± 5.59	14.93 – 21.68	3.02	0.006	1.15
	Experimental	24.38 ± 4.61	21.60 – 27.17			

Summary of findings

Both instructional groups demonstrated improvement in written knowledge following the educational intervention. However, only the Cadaveric Training Group achieved a statistically significant advantage in procedural skill acquisition, reflected by higher checklist scores and a large effect size (Hedges’ g = 1.15).

Proficiency analysis further highlighted this difference. Only 1 of 13 students (7.7%) in the Traditional Instruction Group met the ≥80% procedural competency threshold (≥26/32), compared with 7 of 13 students (53.8%) in the Cadaveric Training Group. This substantial difference supports the hypothesis that incorporating cadaveric model practice into ultrasound-guided regional anesthesia education meaningfully enhances procedural competency beyond lecture- and video-based instruction alone.

## Discussion

Overview

Cadaveric practice produced a large effect (Hedges’ g = 1.15) on procedural skill acquisition, demonstrating a clear educational advantage over lecture- and video-based instruction for teaching the SAPB. While both groups showed similar improvements in written knowledge, the cadaveric training group far outperformed the traditional instruction group in procedural competency, with over half achieving proficiency compared to only one student in the control group.

Educational significance

These findings align with literature supporting cadaveric training as a valuable component in UGRA education. Prior studies have shown that cadaver-based instruction offers realistic tissue feedback and three-dimensional anatomical orientation that enhances the learning of sonoanatomy and needle-handling skills [3-5]. Such experiences are particularly important for novice learners, who may otherwise struggle to translate cognitive knowledge into psychomotor competence using video or simulation alone [6]. This study adds evidence that procedural skill acquisition benefits not merely from passive observation but from hands-on, anatomically authentic practice.

Comparison with previous studies

Several studies have reported improved learner confidence and comfort following cadaver-based UGRA training [3,7]. However, far fewer have evaluated objective procedural performance, making the present study an important contribution to this gap. Our findings align with results from Sites et al., who emphasized structured UGRA training recommendations [6], and from Munirama et al., who demonstrated the enhanced sonoanatomic fidelity of Thiel-embalmed cadavers [8]. These educational advantages likely contributed to the large effect size observed in our study (Hedges’ g = 1.15).

Cadaveric models provide high-fidelity anatomic realism and tactile feedback that help integrate probe handling, sonoanatomy recognition, and needle manipulation-features shown in prior studies to support schema formation and reduce intrinsic cognitive load for novice learners [3-5,8]. Studies comparing cadaveric models with phantoms or low-fidelity simulators similarly report superior learner engagement, improved anatomic understanding, and greater perceived preparedness for clinical UGRA performance [3-5].

Despite promising procedural outcomes, translation of cadaver-based training to live patient performance remains incompletely studied. Most UGRA education research, including this study, assesses learners on cadavers or simulators, and only a limited number of investigations have examined skill retention or clinical outcomes such as analgesic efficacy, opioid reduction, or procedural safety [3,9,10]. A case report has evaluated SAPB clinical outcomes [11], and additional studies further highlight the need for more robust evidence in SAPB implementation and education [12,13]. Performance-based scoring systems described by Niazi et al. also support objective assessment frameworks for regional anesthesia education [14].

Limitations

This study was conducted at a single institution with a relatively small sample size, which may limit generalizability. Procedural performance was assessed on cadaveric specimens rather than live patients; therefore, findings do not directly reflect clinical outcomes. Additionally, evaluators were not blinded to group assignments, which may introduce rater bias. The study also did not assess long-term skill retention or transferability to clinical performance. Variability in cadaveric tissue quality and preservation may affect ultrasound image fidelity and needle visualization. Finally, operational considerations, including cost, facility requirements, and limited scalability, may restrict broader implementation.

Future directions

Future studies should evaluate hybrid curricula incorporating cadaveric training alongside simulation, supervised clinical performance, and mastery-learning paradigms [9-11]. Longitudinal studies are needed to determine whether early cadaveric experience facilitates durable skill retention and improves patient outcomes. Cost-effectiveness analyses may further guide optimal integration of cadaveric resources into medical education programs.

## Conclusions

This study demonstrates that cadaveric model instruction significantly enhances procedural competency among medical students learning the SAPB, compared to traditional lecture- and video-based learning alone. While both groups showed comparable gains in written knowledge, only those with hands-on cadaveric experience achieved substantial improvements in practical skill performance, underscoring the importance of tactile feedback and anatomic realism in developing UGRA proficiency.

Given the significant improvement in procedural competency observed in this study (Hedges’ g = 1.15) and the markedly higher proportion of students achieving proficiency, educators should consider integrating structured cadaveric training sessions into UGRA curricula for novice learners-particularly for complex blocks such as the SAPB. Cadaveric practice provides realistic tissue handling, sonographic fidelity, and reduced cognitive load, which collectively support more efficient skill acquisition than didactic methods alone. Future research should evaluate long-term skill retention, translation to clinical performance, and the feasibility and scalability of implementing cadaver-based training within medical education programs.

## Appendices

Pre- and post-survey questions

Demographics

1. What is your current year of medical training? • OMS-I • OMS-II • OMS-III • OMS-IV • Other: _______

Familiarity & Experience

2. How familiar are you with the serratus anterior plane block (SAPB)? 1 = Not familiar 2 = Slightly familiar 3 = Moderately familiar 4 = Familiar 5 = Very familiar

3. How comfortable are you performing an SAPB? 1 = Not comfortable 2 = Slightly comfortable 3 = Moderately comfortable 4 = Comfortable 5 = Very comfortable

4. Have you ever observed an SAPB performed on a patient? • Yes • No

5. Have you previously watched an instructional video of an SAPB? • Yes • No

Knowledge Assessment

6. Which of the following are indications for performing an SAPB? (Select all that apply) □ Rib fractures □ Thoracotomy pain □ Herpes zoster □ Breast surgery

□ Abdominal surgery □ Appendicitis

7. Which of the following is a contraindication to performing an SAPB? □ Local infection at the injection site □ Coagulopathy □ Allergy to local anesthetic □ Hemothorax □ None of the above

8. Which ultrasound probe is most appropriate for this procedure? A) Curvilinear probe B) Phased array probe C) Linear high-frequency probe D) Endocavitary probe

9. What ultrasound mode is typically used for needle visualization? A) B-mode B) Doppler C) M-mode D) Tissue harmonic imaging

10. Which anatomical landmarks are essential for identifying the injection site? (Select all that apply) □ Midaxillary line □ 5th intercostal space □ Serratus anterior muscle □ Latissimus dorsi muscle □ External oblique muscle □ Xiphoid process □ 10th intercostal space

11. Which artery must be identified to avoid accidental puncture? A) Subclavian artery B) Thoracodorsal artery C) Internal thoracic artery D) Axillary artery

12. The anesthetic is injected between which two structures? A) Pectoralis major and serratus anterior B) Latissimus dorsi and serratus anterior C) Serratus anterior and external oblique D) Serratus anterior and trapezius 13. Injecting too inferiorly may result in which complication? A) Hematoma

B) Pneumothorax C) Infection D) Intercostal neuralgia

14. What is the best way to confirm correct needle placement? A) Loss of resistance B) Visualization of local anesthetic spread under ultrasound C) Aspiration of blood D) Patient-reported paresthesia

Clinical Reasoning

15. In which clinical scenarios would you consider an SAPB? (Select all that apply) □ Rib fractures □ Thoracic trauma □ Postoperative breast surgery pain □ Abdominal pain □ Renal colic

16. For acute rib fracture pain, which treatment are you most likely to choose? A) Oral NSAIDs or opioids B) Serratus anterior plane block C) No treatment D) Unsure
